# Spirituality and Religious Diversity in Nursing: A Scoping Review

**DOI:** 10.3390/healthcare10091661

**Published:** 2022-08-31

**Authors:** Carla Murgia, Ippolito Notarnicola, Rosario Caruso, Maddalena De Maria, Gennaro Rocco, Alessandro Stievano

**Affiliations:** 1 Department of Biomedicine and Prevention, University of Rome Tor Vergata, 00133 Rome, Italy; 2 Centre of Excellence for Nursing Scholarship, OPI, 00133 Rome, Italy; 3 Health Professions Research and Development Unit, IRCCS Policlinico San Donato, 20097 Milano, Italy; 4 Department of Biomedical Sciences for Health, University of Milan, 20122 Milan, Italy; 5 Department of Experimental and Clinical Medicine, University of Messina, 98125 Messina, Italy

**Keywords:** nursing, pluri-religious society, religion, scoping review, spirituality

## Abstract

Spirituality is a common theme in the field of healthcare research. This study aimed to examine nurses’ perceptions of spirituality in the context of the religious diversity of patients in pluri-religious settings. We performed a scoping review following the methodology on studies conducted between 2010 and 2020. We searched the following databases: CINAHL Plus, PubMed, and PsycINFO. For the identification of grey literature, the OpenGrey database was used. In total, 789 articles were reviewed. Of these, 16 met predetermined inclusion criteria. Two main overarching themes emerged from our inductive analysis: (a) the intertwining of spirituality and spiritual care in diverse religious landscapes and (b) obstacles impeding the inclusion of spiritual care in pluri-religious settings. According to our results, nurses consider that spirituality is interconnected with spiritual care for individuals from different religious backgrounds. Interpretations of spirituality in nursing practice vary widely, with spirituality and religiosity often shaped and influenced by culture and the experience of the professionals. Nurses attribute various meanings to spiritual care, most of which center on respecting personal, interpersonal, and relational aspects of religious and cultural beliefs and practices. Lack of education and specific skills, insufficient time, role ambiguity, and different religious beliefs were identified as hurdles to spiritual care. A poor work environment, a lack of patient privacy, including personal space, and a lack of compassion were also reported as deterrents to spiritual healing. More knowledge and training on different religions and spirituality are required to meet patients’ spiritual needs to better overcome these hurdles.

## 1. Introduction

Spirituality is a common theme in healthcare research and on healthcare professionals worldwide, and it constitutes one of the six quality of life domains in the World Health Organization Quality of Life Instrument (World Health Organization, 2012) [1]. Spiritual diversity is widespread due to the existence of pluri-religious societies worldwide. Consequently, nurses commonly care for patients from diverse religious backgrounds. According to the literature, people with and without religious convictions report the need for and benefits of spiritual support in healthcare [2,3,4]. Growing attention to spiritual aspects of care exists even within highly secular societal environments [5]. Worldwide, nurses’ interest in spiritual care is increasing [6,7].

Many studies have shown that nurses consistently recognize the importance of spiritual comfort and value the fundamentals of a holistic approach without any religious and cultural distinction [8,9]. Modern professional nursing arose in a Christian-based values environment and was imbued with religiously derived principles from its beginnings. However, according to the ethical codes of professional organizations and official statements of the International Council of Nurses (2021), holistic nursing must respond to all patients’ spiritual needs, irrespective of their religious beliefs [10]. Nursing spirituality has been at the heart of nursing theory and research for over 30 years. In this framework, the literature reveals that nurses have examined different spiritual perspectives. Stephenson and Hebeshy [11] pointed out that spiritual care should be incorporated into care plans and that nurses need updated knowledge and training on different forms of spirituality [12,13,14]. Other research found that the effectiveness of nursing care depended on nurses’ awareness of and sensitivity to the spiritual needs of their patients [15].

In the contemporary literature, there are three main approaches to spirituality: religious, secular, and holistic [16]. Spirituality is often viewed in broad terms, in which it is defined by the individual and is not necessarily connected to organized religion [17,18]. Worldwide, there is a concerted effort for spiritual assistance and spiritual support to be an integral part of the role of the nurse [19,20]. In clinical nursing practice, it is frequently unclear how to engage patients in spiritual care, especially in complex health systems and societies characterized by secularism and religious pluralism [12,14,21]. Although the role of spiritual care in palliative care and oncology is well recognized, less is known about its role in other areas of nursing, where spirituality and spiritual care are often neglected or absent [5]. Nurses do not consistently integrate patients’ spiritual needs into their daily practice, either because they do not have the time to explore patients’ spiritual demands or because they perceive they lack the skills to provide their patients with spiritual support. In the cultural context of this review, Catholic or Protestant priests or pastors and chaplains or spiritual care providers [22] are the only institutional figures in healthcare settings (hospitals and clinics) that provide spiritual support to patients [12,14,23,24]. However, other professionals also provide spiritual assistance and services, although their involvement may be minor or not recognized. These include nurses [5,23,25], rehabilitation health professionals [26], psychologists, and physicians [27].

Thus far, most studies on spirituality have been conducted in Western countries among homogeneous samples from predominantly Judeo–Christian cultures [5,28,29]. There is a need for studies on spirituality in the healthcare setting, especially nursing, in Asian countries [6,30] and in diverse ethnocultural and practice contexts [5]. The primary aim of this scoping review was to examine studies that focused on the sensitivity of nurses to issues pertaining to spirituality and religious diversity in nursing in Western and non-Western contexts.

## 2. Materials and Methods

### 2.1. Design

This study adopted the methodology of Arksey and O’Malley [31] for scoping reviews, one of the first methodological frameworks to shape this research synthesis. A scoping review is an evidence-based methodology that systematically maps vast bodies of emerging, complex, and extensive evidence (quantitative and qualitative or mixed) to broadly identify data sources and literature gaps [31,32]. For clarity, the Arksey and O’Malley method [31], in conjunction with that of Colquhoun et al. [33], was used. The six-stage methodological structure applied was as follows:

Stage 1: Identification of the research question.

Stage 2: Identification of studies relevant to the research question.

Stage 3: Selection of studies for inclusion in the review.

Stage 4: Charting of information and data in the included studies.

Stage 5: Collection, summary, and reporting of the results.

Stage 6: Stakeholder consultation (optional).

### 2.2. Identification of the Research Question

Our research question aimed to examine what was known about nurses’ sensitivity and understanding of spirituality in the context of patients’ religious diversity in pluri-religious settings. The “population, concept, and context” (PCC) framework has been adopted as per the indication of Arksey and O’Malley [31] to define the research question. In our framework, the population was composed of nurses, the concept was the nurses’ views of spirituality, and the context encompassed all healthcare settings but palliative care.

### 2.3. Identification of Studies Relevant to the Research Question

To shed light on nurses’ understanding of patients’ spirituality, we conducted a literature search of three electronic databases: CINAHL Plus, PubMed, and PsycINFO. The keywords utilized in the literature search included “spirituality”, “diversity religion”, and “nursing”. Boolean operators were employed as conjunctions to merge these keywords in the search [31]. The exact search string used in the search of CINAHL Plus was used to identify grey literature in the OpenGrey database.

All materials were managed via Zotero software, and identical references were eliminated. Three reviewers (CM, IN, and AS) then conducted an iterative two-step screening process of the articles, reviewing first the titles and abstracts and then the full texts, if deemed suitable for further examination. Throughout the process, any disagreement in terms of article selection was resolved by discussion.

### 2.4. Selection of Studies for Inclusion in the Review

This scoping review included papers of different designs (qualitative and quantitative primary research, secondary research, editorials, and commentaries) considering their purpose, which had to be consistent with mapping nurses’ views of spirituality for patients. The extracted data from the included papers were organized considering the aim, design, participants, and context.

### 2.5. Eligibility and Selection Process

Eligible studies were empirical publications that fit the following criteria: (a) qualitative, quantitative, and mixed methodologies; primary and secondary studies; (b) studies written in English and published between 2010 and 2020 to include the most recent research on the topic, as a literature review including papers until 2010 is available [34]; (c) studies with abstracts and full texts, with a focus on religious diversity in nursing care; (d) studies performed in various care settings (hospitals and homes) but not palliative care environments; and (e) studies where nurses were the main surveyed population, followed by patients, caregivers, and other healthcare workers. Studies focusing on palliative care settings were excluded because much of the current nursing spirituality literature focuses on these backgrounds rather than on other types of care settings (e.g., acute care). All studies without abstracts and not written in English were also excluded. Reviews and other types of studies, such as commentaries or analyses of concepts, provided background knowledge of the research problem, and these were read for valuable insights that involved religious diversity but were not included.

### 2.6. Charting of Information and Data in the Included Studies

**Figure 1:**
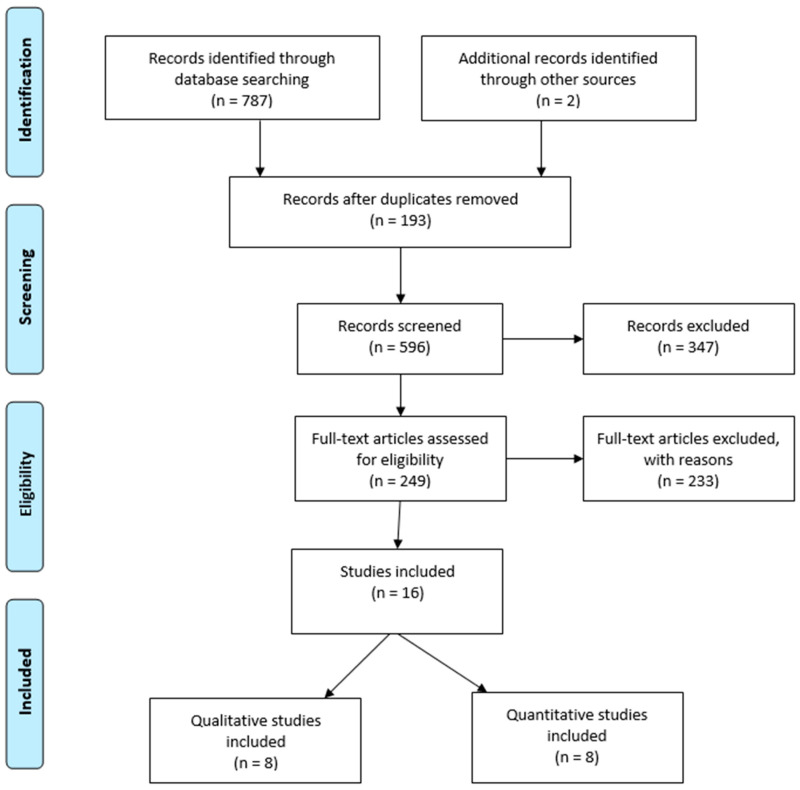
Preferred Reporting Items for Systematic Reviews and Meta-Analyses Extension for Scoping Reviews Checklist (PRISMA-ScR) flow diagram.

Following a literature search, in total, 787 articles were initially selected. The process for item selection is outlined in Figure 1. Reporting was compliant with the Preferred Reporting Items for Systematic Reviews and Meta-Analyses Extension for Scoping Reviews Checklist (PRISMA-ScR) [35]. PRISMA-ScR contains 20 fundamental reporting requirements and two discretionary requirements to include when conducting a scoping study. The publications retrieved described how nurses defined spirituality as interconnected with spiritual care in the context of religious diversity. After meticulously reading all the included research, the synthesis and interpretation of data were performed, and finally, the results were obtained [31].

### 2.7. Collection, Summary, and Reporting of the Results

Although a quality assessment of studies included in a scoping review is not mandatory, we conducted a preliminary assessment of the quality of the included papers using the Critical Appraisal Skills Program checklist [36]. Our aim in doing so was to provide a preliminary background on the included materials that sustained the discussion among authors regarding the evaluation of the studies retrieved. Two of the authors performed this preliminary assessment independently. When they reached a consensus on evaluating the included papers, their evaluations were shared with the entire group of authors. The salient characteristics of the studies were organized as follows: summary of the authors, study location, year, context, study design, number of participants, purpose, analysis, and results.

In line with the purpose of our review, we included studies that focused on the sensitivity of nurses to spiritual needs in Western and non-Western contexts. The data extrapolated from the selected articles were coded using an inductive content analysis [37], extracting significant elements from the information retrieved. Verbatim transcriptions were read and reread to acquire a general sense of the content. Checking the content gathered by the research team members enabled the identification of key concepts. These key concepts were discussed, processed, and reconceptualized into categories describing the sensitivity of nurses regarding spirituality and religious diversity in nursing in Western and non-Western contexts. In this final phase, the categories were divided into two main overarching themes (abductive approach) [38]: (a) the intertwining of spirituality and spiritual care in diverse religious landscapes, and (b) obstacles impeding the inclusion of spiritual care in pluri-religious settings.

## 3. Results

### 3.1. Characteristics of the Included Studies

The reviewed studies were retrieved from those published in Western (e.g., Australia, Canada, UK, and U.S.) and non-Western countries (i.e., Asia and the Middle East). Our results are based on 16 studies, 8 quantitative and 8 qualitative. The studies were published between 2010 and 2020 in the following countries: Canada (*n* = 4), Turkey (*n* = 2), the U.S. (*n* = 2), Australia (*n* = 1), Jordan (*n* = 1), Korea (*n* = 1), Iran (*n* = 1), Malaysia (*n* = 1), Saudi Arabia (*n* = 1), Singapore (*n* = 1), and the UK (*n* = 1). Together, these investigations represented a sample of 3723 participants who comprised nurses, caregivers, other healthcare practitioners, and recipients of care (i.e., patients). The nurses worked in different care settings in various countries (Table 1).

In most of the studies conducted in Western countries, the participants were predominantly Christian nurses. Some of the studies on spirituality mentioned other religions, such as Buddhism and Hinduism [13]. Islam was rarely cited [13]. As reported in Table 1, the included studies assessed nurses’ perceptions of spirituality and spiritual care in association with socio-demographic characteristics [6,7,8,13,39,40,41,42,43], various hospital settings [6,7,12,22,41,42,43,44], acute care [6,24,37,44], home care [45], and their involvement in spiritual care practices [6,7,13,22,24,40,42,44,45]. Many of the studies used validated tools to explore the spiritual observations of the nurses [7,13,29,43].

Heterogeneous samples of nurses from different ethnic and religious backgrounds considered spirituality from different points of view [6,7,12,13,22,24,41,45,46,47]. Nurses, caregivers, patients, family members, administrators, and other practitioners were included in only three qualitative investigations [22,39,45]. In one 4-year study, religious diversity was considered through the concept of shared sacred space [39]. Four studies explored perceptions of spirituality in a sample of nurses with the same religious affiliation [5,40,42,48]. These studies focused on nurses from Arab cultural and religious backgrounds [5,40,42,48]. Only one study provided nursing skills development guidelines to help non-Muslim nurses in Saudi Arabia understand the needs and preferences of Muslim patients [11].

Based on the findings of most of the studies, nurses consider spirituality to be pivotal in nursing [6,7,8,13,22,24,28,39,40,41,42,43,44,45,46] and attribute various meanings to spirituality and spiritual care, most of which are centered on their religious beliefs [6,7,8,13,22,24,28,39,40,41,42,43,45,46]. However, there was some confusion regarding the meanings of spirituality and spiritual care [6,7,12].

Generally, spiritual care was influenced by the individual’s religious identity [7,12,22,28,45], the specific healthcare organization [7,12,24,28,39,42,44], and the reference environment [6,7,8,12,22,39,42,45]. There seemed to be broad agreement that spirituality and spiritual care education had to be integrated into university teaching programs and continuing education [41,43,44]. For example, concerning the healthcare setting, the results indicated that the workplace, such as the type of structure, religious or nondenominational [7], or private or governmental [40], influenced nurses’ practices concerning spirituality and spiritual care. Healthcare organizations that operated within economic constraints created barriers to spiritual care provision [22,39]. Several obstacles to the practice of spiritual assistance emerged, the main one being time constraints [6,12,22,24,39,41,42,44,45], followed by a shortage of personnel, fear of crossing professional boundaries, fear of proselytism [22,24,28,45], and difficulty in recognizing spiritual needs when spiritual beliefs differed from those of the care provider (nurse/healthcare institution) [41,44]. However, most surveyed nurses in all clinical areas provided spiritual assistance as a personal creed and as part of their clinical practice. Elements of spiritual care included a range of behaviors, such as sitting with patients in silence, praying with them, and respecting privacy [7,13,22,24,39,41,42]; listening, giving, and receiving comfort; or communicating with the healthcare chaplain or spiritual assistant, regardless of the religion of the patient or affiliation with particular religious bodies. [6,22,24,39,42,44].

### 3.2. Intertwining of Spirituality and Spiritual Care in Diverse Religious Landscapes

Spirituality has long been part of nursing and represents an essential value for nurses, patients, and their families. Spirituality is intertwined with spiritual care, which can enhance health outcomes [6] and is part of a holistic approach to medicine [39,41,46,47]. Spirituality is manifested uniquely in each individual and among different religions or spiritual groups (e.g., Buddhism, Christianity, Hinduism, Islam, Judaism, and others). Spirituality is considered an essential aspect of life in all world cultures; it is a dimension of the person that nurses in Western, Middle Eastern, and Asian countries respect [8,48].

Cumulative evidence points to a positive contribution of spirituality to the health and well-being of the individual, family, and community [18,47,49,50], and spirituality plays a significant role in stressful work circumstances [51]. Nevertheless, nurses’ interpretations of spirituality in nursing practice vary widely [4,47]. Spirituality is an umbrella term covering an extensive array of personal meanings, interpretations, and associations [28]. Nurses likened spirituality to a unifying “force” that enables patients to seek peace, meaning, and resolution in life during periods of healing from illnesses [52].

In many societal contexts, spirituality is interpreted as religion [6,40]. Spirituality is often understood as the antithesis of religiosity [45]. Today, the dominant forms of spirituality are Eastern or Western [53,54]. However, the two forms overlap and have points in common [21,55]. Spirituality is primarily influenced by religion, culture, societal pluralism, history, and personal perspectives [18,38,47,48,56,57]. Nevertheless, there is little clarity and consensus on spirituality’s meaning [18,38,47]. One definition of spirituality contends that spirituality is a dynamic and intrinsic aspect of humanity through which persons seek ultimate meaning, purpose, and transcendence, and experience relationships to self, family, others, community, society, nature, and the significant or sacred. Spirituality is expressed through beliefs, values, traditions, and practices [18,58].

Religious diversity is a term used to indicate the existence of many religious traditions in a multiethnic, multicultural society. Our secular society, which is becoming progressively more globalized, is characterized by increased spiritual diversity. Such diversity is growing in many communities, with immigrants contributing significantly to this diversification [22,39,45,50,54,59]. The religious landscape in the Western world differs from that in other parts. In the West, membership of (Christian) religious organizations or groups has remained stable or decreased. For example, church membership in the United States decreased from 67% in 1996 to 32% in 2015 [58]. However, religion remains a potent force in Canada, although no officially recognized state religion exists. In fact, the influence of Christianity (both Protestantism and Catholicism) remains strong at the societal level [22,59]. In Europe, the leading Christian religions, particularly Catholicism, Orthodoxy, and Protestantism, are the most widespread. As of 2018, about 70% of the European population identified as Christian in some form [60]. Nevertheless, in the U.S., the population, both Christian and unaffiliated, is noticeably more religious than their European counterparts [18,61].

Beyond a single institutional religion (Christianity) in Western countries and the European continent, religious diversity reflects growth in new spiritualties, with different spiritual expressions in many societal contexts, especially among people who identify as “spiritual but not religious” [14,18,22,39]. The division between religion and spirituality is more difficult to perceive in the Middle East. Furthermore, in the Middle East, nonreligious terminology is uncommon [48], and spirituality is inseparable from religion, as it derives from the Holy Quran [8]. Confucianism, Buddhism, and Daoism do not qualify as religions because they are not organized and formal [30].

From a Middle Eastern religious perspective, there is no distinction between spirituality and religion [8,40,42]. It is part of the culture and beliefs of the Middle East to pray several times a day, even during working hours. For most Muslims, religiosity pervades all aspects of their daily lives, including their working lives [13,48]. Spirituality in a Middle Eastern context is crucial and is founded on respect for Islamic religious beliefs and the values of persons [7,40,42,48]. Islamic spirituality is regarded in terms of the relationship with Allah [7,11]. Of the studies included in this review, one study, that of Stephenson and Hebeshy [11], provided brief guidelines on the principles and practices of Islam to help non-Muslim nurses in Saudi Arabia understand the needs and preferences of Muslim patients. In terms of spiritual care and religion, many nurses, who identified with the Islamic religion and prayed at work every day, attributed the utmost importance to patients’ religious needs, considering the fulfilment of these needs an ethical obligation and commitment. Weathers [48] compared perspectives on spiritualism in the Middle East to those in the West. Similar results were reported in studies conducted in Turkey [43,46], Jordan [9], and Iran [42]. The separation of spirituality from religion in Western thinking is recent and is the result of sociological disengagement from religious organizations. In this framework, to ponder spirituality as a residual religion rather than as some universal entity or substance is essential to comprehend all human beings everywhere. Places such as Saudi Arabia, where such societal disengagement has not occurred, do not require a separate category.

Nurses are occasionally unaware of these sociological aspects and sometimes consider patients as belonging to one faith, especially in Western countries where Christianity predominates, now and then silently influencing care with its creeds. Practicing deep listening and encouraging patients to discuss spiritual or existential issues, sharing prayers or songs [47,52], referring patients to a chaplain or a spiritual leader [30], and using religious texts for Muslims [42] can strengthen the nurse/patient relationship and provide spiritual comfort to patients. However, religious diversity and secular issues may give rise to conflict among care providers regarding the appropriateness of praying [52].

As mentioned, nurses sometimes do not have the sociological background to comprehend the different nuances of religious diversity fully. However, according to them, spiritual care involved empathic discourse, and empathy was regarded as a personal and profound form of expression associated with spirituality [7]. Canadian ethnographic studies highlighted the roles of religion and spirituality. These studies included diverse populations in terms of ethnic, religious, and spiritual affiliations (Christian, Sikh, none, Muslim/Islamic, atheist, Hindu, Greek Orthodox, and others); care settings (medicine and nephrology); and organizational settings (community hospitals, home care, and hospices) [22,45].

Spirituality and spiritual care have been interpreted as mutually beneficial. Spiritual care could take the form of preparing a patient to attend their temple [45] (p. 20). Respect for others and privacy, dignity, and support for the culture and beliefs of individual patients, together with compassion, kindness, and joy, are the cardinal principles of care interpreted as spiritual [13,14,39]. For example, a healthcare practitioner can show respect for a patient during a home visit by recognizing religious differences via the presence of symbols and signs (e.g., home altars featuring Hindu statuettes, Buddha icons, or crucifixes). These are crucial factors that are not usually achieved in hospitals.

The role of nurses in managing religious diversity is multifaceted. In-home caregivers and health workers may come from a variety of religious or nonreligious backgrounds (agnostics and atheists) and hold spiritual beliefs or not. This was evident in one homecare patient’s comment: “the world comes to my home” [45] (p. 15). Here, the patient was referring to Filipino migrant workers. As clear in various studies, individuals in need of care, whether home care or hospitalization, attach great significance to having their emotional (e.g., kindness, humor, and friendship) and spiritual needs met [22,41].

In previous research, Reimer-Kirkham [14] conducted a critical analysis of religion, politics, nursing, and healthcare and demonstrated the role of religion and spirituality in health institutions and how health institutions were committed to respecting different forms of religion. Importantly, Reimer-Kirkham [14] reflected on how spirituality related to religiosity in different social contexts and how it was identified and addressed by health professionals and nurses. Reimer-Kirkham et al. [39,45] focused on studies in which religious and ethnic diversity received equal attention from healthcare managers, caregivers, and care recipients. They examined the negotiation of the dynamics of religious, spiritual, and ethnic plurality in hospital and home health services and analyzed how social, economic, and political contexts and gender shaped these dynamics. Religion and spirituality intersected with class relationships, creating tension and marginalization rather than connection [22]. However, in other settings, there was an intimate exchange between the patients and health professionals who explored the complexity of diversity in homecare [39,45]. Reimer-Kirkham et al. [45] stated that what united the interviewees was their shared religious identity, which led to comfort and connection. The “religion taboo” seemed to disappear [45]. The nurse became the guardian of care [12] and the provider of emotional support [22].

A Korean [24] and a Singaporean [7] study selected a heterogeneous sample of respondents for consistency with diversity. The Korean research considered the religious diversity of nurses with different opinions, perspectives, and experiences as inclusion criteria to ensure homogeneity within groups and heterogeneity and effective communication among groups. Clinical practice and religion strongly exerted influence on spiritual healing [24]. Always in the context of religious diversity, Cooper et al. [47] explored the social and power characteristics underlying nurses’ communication. The participants were a group of 20 nurses, with 14 nurses employed in a private religious hospital managed by a religious body and 6 nurses employed in a nondenominational hospital. The sample comprised Christian and Islamic nurses and those without religious affiliations. The nurses worked in different settings, had different years of experience, and had diverse socio-demographic, cultural, and religious backgrounds. The selection of a sample with these characteristics allowed for a broader vision of how nurses conceptualized spirituality based on religious differences. The first critical discourse that emerged from the interviews was personal religious belief, followed by other beliefs and faiths [7].

#### Instruments to Measure Spirituality

Chew et al. [7] used Tiew and Creedy’s Spiritual Care-Giving Scale (SCGS) [62] to explore possible associations between the personal and professional characteristics of nurses and their perceptions and experiences of spirituality and spiritual care in an acute-care hospital in Singapore, a multicultural and pluri-religious city-state. Their findings showed that nurses expressed positive perceptions about spirituality and spiritual care, making themselves available to provide spiritual care through an interprofessional collaborative approach involving other nurses, clergy (e.g., chaplains), and leaders of other religious groups, thereby enabling a broader, more inclusive perspective. However, nurses were often unclear on the meaning of spiritual care. The high participation rate (76%) and the diversity of the multiethnic (Indian, Chinese, Malaysian, and others) and pluri-religious (Christian/Catholic, Islamic, Hindu, and other affiliations) sample constitute strengths of their study. In the study by Chew et al. [7], the SCGS scores were statistically significantly associated with three variables: the area of clinical practice, religion, and the perception of the spiritual self.

In line with the study by Chew et al. [7], Atharim et al. [13] reported that nurses in Malaysia navigate a society characterized by religious diversity. Official religions recorded in Malaysia in 1965 included Islamism (61.3%), Buddhism (19.8%), Hinduism (6.3%), Christianity (9.2%), and atheism (0.8%), with followers of the indigenous faith recorded more recently [7,13,45]. Atarhim et al. [13] used the Spirituality and Spiritual Healing Rating Scale (SSCRS), translated into Malay and applied it in a Malaysian professional context. They obtained similar results to those of studies in other countries, such as in Turkey by Akgün Şahin and Ozdemir [43]. Their study provides preliminary information on spirituality and spiritual care from the nurses’ perspectives. More than 90% of the respondents were Muslim, and the remainder identified as Buddhist, Christian, and Hindu. Although the nurses provided spiritual care because this is required in line with their religious beliefs and societal norms, they pondered whether spiritual care could be provided to nonreligious individuals, such as atheists and agnostics [13]. In the study, higher education, marital status, older age, more years of work experience, longer working hours, medical department work, and spiritual care training were positively associated with higher SSCRS scores [13].

### 3.3. Obstacles Impeding the Inclusion of Spiritual Care in Pluri-Religious Settings

Lack of education in the area of spiritual care and skills specific to this field of care [28,43], insufficient time, role ambiguity [12,13,22,24,42,43,44], economic constraints [22,59], and different religious beliefs of nurses [27] were identified as the main hurdles to spiritual care. Another significant barrier to the provision of spiritual care arose from the absence of a shared definition of spirituality between providers and patients, contributing to embarrassment and confusion about what spirituality and nurses’ spiritual care practices comprised. Furthermore, it was not clear how spiritual care differed from nursing in general [11]. In addition, busy and noisy work environments and insensitivity to compassion due to space constraints [55] were reported as deterrents to spiritual healing [24,39]. Other barriers revolved around ethical aspects of spiritual care [23] or making patients feel uncomfortable about spiritual care [22,24,55]. In addition, the difficulty in distinguishing spiritual care from proselytism [24,55], the fear of criticism from others [6], and the lack of a registration system for preferences regarding spirituality [24] were cited as obstacles to the provision of spiritual care. These factors were considered possible hindrances that created doubt or confusion among nurses and patients regarding spiritual care choices. Most nurses cited the need for more education and training on the spiritual aspects of care delivered to different ethnic groups [24]. Education is needed to enhance nurses’ awareness of religious and spiritual diversity and to enable them to meet the spiritual needs of their patients [6,41].

At the same time, spirituality has to be addressed within the nursing curricula, ensuring that the practice of spiritual care is not neglected [6,13,46]. Regarding the inclusion of spirituality at a practical level in care, three categories emerged: personal influences, organizational influences, and social influences.

#### 3.3.1. Personal Influences

The findings of several studies suggested that definitions of spirituality and spiritual care were expressed according to the nurses’ beliefs [39,41,44]. Although this finding was robust among participants of Christian origin, for Muslims, there was no distinction between religion and spirituality, with spirituality an inherent part of daily life. All aspects of their daily lives were guided by faith and rituals [11,42]. Discourse on personal religious beliefs molded spirituality. Nurses were influenced by their religious attitudes or by the religious perspectives of their family, even for those who were not religious. Talking about spirituality meant using religious terms. For example, spirituality often coincides with personal religion. Consequently, nurses viewed their religion as a guide and a motivation, thus influencing the spiritual care they provided [28,44]. Another influencing factor was conceptual ambiguity, consequently making it a conditioning or a marginalizing factor among nurses [24,55].

#### 3.3.2. Organizational Influences

Nurses’ attitudes to caring strongly influenced limiting or making possible the practice of spiritual care. The type of organizational structure (religious or nondenominational) affected nurses’ attitudes to spiritual care provision, university training, and the work environment. Despite its influence and relevance, Reimer-Kirkham et al. [45] concluded that religion was like the “elephant on the table” because it was stated that it was not a nursing competence. A similar conclusion was reached in the study by Janzen et al. [12], in which, in a temporal context of diversity, spirituality was recognized as precious, but it was added that “for spirituality it would have been necessary to spend a little more time, to make an extra effort, but this did not happen because it was airy-fairy. Therefore, pushing it under the carpet was easier to avoid offending anyone” [12] (p. 256). In this situation, nurses preferred not to be involved in spiritual care. Sometimes, spirituality was considered not to be a nursing competence.

Lack of institutional support, time, and sufficient staff to provide comprehensive care and a heavy workload were frequent problems that hindered spiritual care [40,41]. The professional boundaries between nurses and chaplains in healthcare settings constituted another hindrance. Blurring boundaries regarding roles could lead to conflict among healthcare professionals [23,41]. Nurses considered the risk of proselytism another potential barrier to the provision of spiritual care [24,28,41,55].

To conclude, in the narratives of the nurses working in private and religious healthcare settings regarding spiritual care provision, nondenominational public hospitals were more guided by the local culture and the values of openness [12].

#### 3.3.3. Social Influences

From a critical analysis of 20 interviews at two hospitals by Cooper et al. [8], one nondenominational public and one private religious hospital, social characteristics and implicit religious power emerged, consequently affecting spiritual assistance and the nurse–patient relationship. In terms of religious diversity, recognizing spirituality as an integral part of the person is a goal of health services. Understanding the social context and how social and cultural–religious factors intertwine, for example, socioeconomic status with environmental factors, can enable nurses to identify factors impeding or facilitating the development of spiritual skills and improving these skills [12,39,45].

Over the past century, secularism has permeated healthcare provision. This might be due to religious diversity or a lack of religious conviction among the people healthcare organizations serve. In this environment, some respondents expressed fear of providing spiritual care [28,39]. In globalized health systems, nurses often consider caring for multifaith patients problematic and uncertain.

## 4. Discussion

This scoping review aimed to identify and summarize nurses’ perceptions of spirituality in the context of the religious diversity of patients in Western and non-Western settings. Sixteen articles met the preset inclusion criteria and were retrieved and analyzed in depth. The results revealed that research on spirituality and health has been predominantly conducted in Western countries on the Christian religion. In Middle Eastern countries, research has focused exclusively on the Islamic religion. This analysis indicated that nurses generally consider spirituality interconnected with spiritual care and the fundamentals of nursing. It also showed that the importance of spirituality in nursing is recognized in professional standards worldwide [10,19,63].

According to some studies, patients find comfort in rituals, prayers, conversations with supernatural entities, and the diligent attention healthcare professionals provide [12,22]. This support, through respect for religion, has been considered engrossing, as spirituality and religiosity are often shaped and influenced by culture [18] and the experience of the care provider [17]. Providing person-centered spiritual care is an essential dimension of dignified care in multicultural settings. This review shows that nurses attributed various meanings to spirituality and spiritual care, mostly centered on respecting personal, interpersonal, and relational aspects of religious and cultural beliefs and practices. Thus, the nurses adopted a personal position based on their identity and responded to the challenge of integrating spiritual assistance into a spiritually diverse landscape according to their personal, organizational, and social positions, which were also shaped by their education, age, years of clinical experience, and workplace environment [12,21,22,47].

In a multifaith society, recognizing spirituality as an integral part of the person and a fundamental goal of health services is vital to comprehend the factors hindering or facilitating the provision of spiritual care [14]. This is particularly true in Western healthcare settings attempting to provide spiritual assistance to patients from various cultural backgrounds. For example, most research in the U.S. defines spirituality according to Christian principles, and there is a lack of information regarding other faiths’ spirituality [11].

In Western and Middle Eastern countries, health organizations are (historically) rooted in a specific religious tradition. In some of these countries, aides known as holy assistants from the same religious background as the patient provide spiritual care. However, religious diversity must be considered when caring for patients from various cultures and religions. Reconceptualizing the meaning of spirituality in healthcare settings and pluri-religious societies is an ongoing challenge [14,19,60]. Through this scoping study, a continuum that extends from resistance and avoidance at one end to deep commitment and responsible pluralism at the other was identified, corresponding to different attitudes among nurses to spiritual care provision, mirroring those of a secular society [12].

At the practical level of care provision, the disconnect between the perceived importance of spiritual care and poor provision of care is explained by several barriers identified by nurses [12,14,39,53]. From the nurses’ perspectives, numerous and distinct factors can hamper the provision of spiritual care, all of which deserve deep reflection. According to Reimer-Kirkham [14], there must be full awareness regarding the concept of spirituality to allow it to be applied and utilized correctly in the discipline of nursing. There are also practical obstacles to the application of spiritual care, such as a lack of institutional and organizational support, a lack of adequate training, and a Christian-centered approach to care in Western healthcare settings, where priests or chaplains provide spiritual care, regardless of the religious beliefs of the patient [22,43]. In these settings, spiritual care provision by non-Christian figures is notably absent [57]. The aforementioned poses a challenge to nurses and could provoke resistance to spiritual care provision, as such care is not one of their competencies. Nurses may also fear misunderstanding the concept of diversity or influencing care recipients with personal beliefs [13,21,63]. The following provides an example of the problems that can arise when religious diversity and personal beliefs are not respected/understood. Hindu family members, on the death of their mother, asked for respect in the management of mourning according to Hindu rites. Instead of contacting a representative of the Hindu religion, as requested by the patient’s family, the nurse called on the services of a Roman Catholic priest [18].

In some cases, patients’ religious and spiritual beliefs may be vital in decisions relating to their health [22,54]. Knowledge of the principles underlying such beliefs is necessary to provide adequate patient-centered care. A trust-based relationship between healthcare professionals and patients is essential to realizing spiritual care. Trust is necessary to help patients and their families to use their resources in the healing or grieving process, irrespective of their personal or professional beliefs or traditions and those of the healthcare organizations [8,59].

The findings of this review indicate that most of the participants received no training related to spiritual care and felt insufficiently trained, despite recent attention on the spiritual dimensions of nursing [13]. To overcome this problem, more comprehensive training on different religions and spirituality is needed to improve nurses’ knowledge in these areas and better meet patients’ spiritual needs [13,26,39,45]. Nurses need to be aware of individual differences and expand their ability to relate to patients of different cultures, faiths, and colleagues. Achieving these goals requires in-depth advanced skills and lifelong learning [18].

In conclusion, nurses need to be aware of patients’ beliefs to ensure that they provide care that is culturally, religiously, and appropriately for the individual care recipient [8,18,22]. Nurses must speak and listen to patients without prejudice and provide personalized and impartial care with the utmost compassion and sensitivity. Knowledge of the basic tenets of these cultures is a prerequisite to helping patients utilize their internal resources in the healing process, irrespective of the nurse’s personal beliefs.

## 5. Limitations

This review included only studies published in English between 2010 and 2020, and it does no justice to the complexity of spirituality and religious diversity in nursing expressed by manuscripts written in other languages. Moreover, it was difficult to delineate clear boundaries among the concepts of religion, spirituality, and spiritual care practices because they are strongly intertwined. Some details that refer to a specific term could have been blurred during the analysis. Moreover, this review did not explore spirituality as residual religion in detail. In this regard, more search terms could have been used to elicit additional studies, especially in grey databases, to achieve a more precise overview of the topic. Furthermore, although we searched a number of well-known health databases, we omitted some sociological and religious studies’ databases. Nevertheless, the qualitative and quantitative papers included in this scoping review allowed for a better understanding of the topic.

## 6. Conclusions and Relevance for Clinical Practice

As revealed in this scoping review, the concept of spirituality is interrelated with spiritual care, and it is an essential and complex concept that has not reached full maturity in the perception of religious diversity in nursing. This review demonstrates that integrating spiritual care into healthcare in a highly pluralized and spiritually diverse landscape is challenging and that there is no single way to address this issue. There are different points of view on integrating spiritual care in healthcare [21]. In this respect, it is necessary for the future to adopt an eclectic approach. Such an approach is critical if the definition of spirituality and the provision of spiritual care is to be inclusive and embrace diversity within society [29].

This review highlights significant challenges and concerns regarding the provision of spiritual care in the nursing profession. These challenges also include severe hospital understaffing, difficult work conditions, and stress and burn-out, which can further hinder spiritual care provision. The knowledge gained from this review may be helpful for nurses to reflect on their position in addressing the spiritual needs of an increasingly diverse patient population in clinical practice. All nurses need to be fully aware of and know the different ways to express spirituality and spiritual care. Through better training in world religions, possibly via postgraduate courses, nursing educators can help to foster continuing education on the topic. Lastly, for hospital managers and policymakers to make informed, bias-free decisions on organizing or implementing spiritual care in healthcare settings, fostering excellence in the provision of spiritual care and nurses’ training in this area should be a priority [64].

## Figures and Tables

**Table 1:** Summary of the included studies.

Author, Year, Location	Context	Study Design, Instruments	Number of Participants	Aim	Analysis	Results
Abdollahyar, et al., 2019 (Iran)	An educational hospital affiliated with Kerman University (noncritical vs. critical care).	A cross-sectional design. The Spirituality and Spiritual Care Rating Scale was used (23 items)	125 nurses	To determine nurses’ attitudes toward spirituality and spiritual care in an educational hospital in Iran.	To analyze data, descriptive statistics were used. The Kolmogorov–Smirnov test was conducted to indicate that data were sampled from a population with a normal distribution. The correlation between demographic data and spirituality and spiritual care mean score was examined by the Pearson and Spearman correlation coefficients, *t*-test, and one-way ANOVA, using the (SPSS Version 21.0. Armonk, NY, USA: IBM Corp).	A significant association was found between nurses’ spirituality/spiritual care attitudes and age, education level, and type of hospital ward employment (noncritical vs. critical care). Nurses’ scores on attitudes toward spirituality and spiritual care suggest the need for more education in this area.
Atarhim et al., 2018 (Malaysia)	All nurses from the Malaysian Nurse Forum Facebook closed group.	An online survey A descriptive cross-sectional study design. The Spirituality and Spiritual Care Rating Scale (SSCRS) (McSherry, Draper, and Kendrick, 2002). (17 items).	208 nurses	To explore Malaysian nurses’ perceptions of spirituality and spiritual care.	The Malaysian Nurse Forum Facebook closed group was used for data collection with 208 completed questionnaires. The Qualtrics software was utilized.	The participants considered that spirituality is a fundamental aspect of nursing. Half of the respondents were uncertain regarding the use of the spiritual dimension for individuals with no religious affiliation. Most nurses felt that they required more education and training relating to spiritual aspects of care, delivered within the appropriate cultural context.
Chew et al., 2016 (Singapore)	Acute care hospital.	A cross-sectional, exploratory, nonexperimental study. Spiritual Care Giving Scale (SCGS Tiew & Creed, 2012), 35 items.	767 nurses	To investigate acute care nurses’ perceptions of spirituality and spiritual care and relationships with nurses’ personal and professional characteristics.	Descriptive statistics and General Linear Modelling were used to analyze data. SPSS 20.0 Mac version (SPSS Inc., Singapore city, Singapore) was used for data analysis.	Acute care nurses reported positive perceptions of spirituality and spiritual care. Religion, area of clinical practice and view of self as spiritual were associated with nurses’ reported perspectives of spirituality and spiritual care.
Cooper et al., 2020 (Australia)	A nondenominational public hospital and a faith-based private hospital.	Qualitative Critical discourse analysis.	20 nurses	To uncover how nurses construct their understanding of spirituality and practice of spiritual care.	Data were analyzed following the qualitative critical discourse analysis procedure proposed by Schneider.	Three discursive constructions of spirituality were identified: personal religious beliefs, holistic discourse, and empathetic care discourse. The work environment had an influence too.
Deal & Grassley 2012 (USA)	Acute and chronic hemodialysis settings.	Phenomenological design.	10 nurses	To explore the lived experiences of nephrology nurses giving spiritual care in acute and chronic hemodialysis settings.	Data were analyzed using Colaizzi’s phenomenological approach.	Five themes were identified: (a) drawing close, (b) drawing from the well of my spiritual resources, (c), sensing the pain of spiritual distress, (d) lacking resources to give spiritual care and, (e) giving spiritual care is like diving down deep
Lee & Kim 2020 (Korea)	Acute care hospital.	Qualitative analysis based on focus groups.	24 nurses	Analyze the experiences of acute care hospital nurses’ on spiritual care.	Data were analyzed following the qualitative content analysis procedure proposed by Graneheim and Lundman.	Five categories with 14 sub-categories emerged: (1) ambiguous concept: confusing terms, an additional job; (2) assessment of spiritual care needs: looking for spiritual care needs, not recognizing spiritual care needs; (3) spiritual care practices: active spiritual care, passive spiritual care; (4) outcomes of spiritual care: comfort of the recipient, comfort of the provider; and (5) barriers to spiritual care: fear of criticism from others, lack of education, lack of time, space constraints, and absence of a recording system.
Gallison et al., 2013 (USA)	The units included oncology, critical care, geriatrics, and the general medical units at an 800-bed academic medical center in New York City.	An explorative, descriptive study. The Spiritual Care Practice (SCP) Questionnaire (Vance, 2001).	120 nurses	To identify barriers in providing spiritual care to hospitalized patients.	Data were analysed using SAS software program, version 9.2	The most common perceived barrier identified was insufficient time, then privacy religion, then difficulty distinguishing proselytizing from the delivery of spiritual care.
Janzen et al., 2019 (Canada)	Various practice settings across the healthcare continuum.	Qualitative secondary analysis.	14 nurses	To explore the perspectives of nurses regarding influences on spiritual caregiving in nursing practice.	Data were analysed using content analysis. In keeping with the method of secondary analysis, transcripts were sorted to (1) fit with the secondary research questions, (2) achieve detailed description of the phenomenon of interest, and (3) provide maximum variation in the data.	Three nested themes were identified as influencing spiritual caregiving: the nurse as custodian of spiritual caregiving, the influence of practice environments and the social context.
Kaddourah et al., 2018 (Saudi Arabia)	Five tertiary care hospitals in Riyadh (Saudi Arabia).	A cross-sectional study design. The Spirituality and Spiritual Care Rating Scale (SSCRS) (Mcsherry, Draper, and Kendrick, 2002). (17 items).	978 nurses	To identify the perceptions towards spirituality and spiritual care.	Data were analysed using SPSS Statistics (Version 23.0. Armonk, NY, USA: IBM Corp).	The participants believed that spirituality exists in all religions and spiritual care means showing concern while serving the patients and focusing on respecting patients’ religious beliefs.
McSherry & Jamieson 2013 (UK)	Members of the Royal College of Nursing practicing in nursing throughout the United Kingdom.	An online survey Open-ended questions in association with a quantitative survey. SSCRS plus open-ended questions, (17 items).	2327 members of the Royal College of Nursing.	To provide an opportunity for members to express their understandings of spirituality and spiritual care.	Content/thematic analysis. Responses to the survey were automatically collated using the ProQuest platform. A retrospective analysis of the qualitative data was also undertaken. The length of answers provided ranged from ‘no comment’ to extensive descriptions of spirituality and spiritual care.	Five broad themes emerged: (1) theoretical and conceptual understanding of spirituality, (2) fundamental aspects of nursing, (3) notion of integration and integrated care, (4) education and professional development and, (5) religious belief and professional practice. Findings suggest that nurses have diverse understandings of spirituality and the majority consider spirituality to be an integral and fundamental element of the nurses’ role.
Melhem et al., 2016 (Jordan)	Four main sectors; University Affiliated Hospitals, private hospitals, governmental hospitals affiliated to the Ministry of Health and military hospitals affiliated to the Royal Medical Services.	A cross-sectional descriptive study.	408 nurses	To describe nurses’ perceptions of spirituality and spiritual care in Jordan, and to investigate the relationship between nurses’ perceptions and their demographic variables.		Most of the participating nurses had a high level of spirituality and spiritual care perception. Significant differences were found between male and female nurses’ perceptions of spirituality and spiritual care (*p* < 0.05); previous attendance of courses on spiritual care also made a significant difference to perceptions (*p* < 0.05).
Pesut & Reimer-Kirkham 2010 (Canada)	Palliative, hospice, medical and renal inpatient units at two tertiary level hospitals and seven community hospitals.	A qualitative study: critical ethnography.	20 health care professionals (nurses, doctors, social workers and other allied health professionals), 17 spiritual care providers (both paid and volunteer), 16 patients/families, and 12 administrators.	To analyze the negotiation of religious and spiritual plurality in clinical encounters, and the social, gendered, cultural, historical, economic and political contexts shape that negotiation. (1) describe how religious and spiritual plurality is negotiated in health care provider/recipient encounters. (2) examine how health care contexts shape the negotiation of religious and spiritual plurality and, (3) critically examine the ways in which societal contexts shape the negotiation of religious and spiritual plurality in health care. The findings pertaining to the first objective, the negotiation of religious and spiritual plurality in clinical encounters.	Data collection, management and analysis occurred concurrently. Interviews were audio-taped and transcribed verbatim. Transcripts and field notes were entered into the NVIVO QSR qualitative software program for analysis.	Clinical encounters between care providers and recipients were shaped by how individual identities in relation to religion and spirituality were constructed. Importantly, these identities did not occur in isolation from other lines of social classification such as gender, race, and class. Negotiating difference was a process of seeing spirituality as a point of connection, eliciting the meaning systems of patients, and creating safe spaces to express that meaning.
Reimer-Kirkham et al., 2017 (Canada)	Home health care	Qualitative analysis-based interview, participant observation and focus groups.	46 participants. Health care providers, administrators, clients.	To explore how caregiver/recipient identities are constructed in home health settings. (2) describe how religious, spiritual, and ethnic plurality is negotiated in caregiver/recipient encounters in home settings. (3) examine how home health services shape how religious, spiritual, and ethnic are negotiated; and, (4) analyze how social contexts shape the negotiation of religious, spiritual, and ethnic plurality in providing home health services.	Data were transcribed and entered Nvivo, a qualitative data analysis program.	The viewpoints of administrators, clients, and healthcare providers traced a pattern of spiritual and managing differences in the provision of home health. These viewpoints were mediated by commonplace constructions of religion and ethnicity in Canadian society and the political economy of home health.
Reimer-Kirkham, et al., 2012 (Canada)	Two hospitals: palliative, hospice, medical and renal inpatient care units within two tertiary hospitals and seven community hospitals.	Ethnographic method	The 55 participants described their religious affiliations as Christian (*n* = 35), Sikh (*n* = 10), Muslim (*n* = 2), First Nations (*n* = 2), Atheist (*n* = 2), Jewish (*n* = 2), Hindu (*n* = 1) or Greek Orthodox (*n* = 1).	To examine the negotiation of religious and spiritual plurality in health care with specific objectives to: (i) describe how religious and spiritual plurality is negotiated in healthcare provider/recipient encounters; (ii) examine ways in which healthcare contexts shape the negotiation of religious and spiritual plurality. (iii) critically analyze ways in which spatial and societal contexts shape the negotiation of the religious and spiritual plurality in health care, and (iv) facilitate knowledge translation into practice, health policy and education.	Data were transcribed and entered Nvivo, a qualitative data analysis program.	The sacred takes form in social and material spaces in hospitals. Sacred spaces included designated ecumenical spaces (formerly called ‘chapels’) and informal sacred spaces created elsewhere (e.g., patient’s bedside). Sacred spaces also involved metaphysical (e.g., a sense of the divine when stepping out into a starry night after attending the death of a patient) or relational (e.g., through interpersonal connections) contexts. These spaces evoked a feeling of a sacredness of space and time—A sense of transcendence, immanence or connectedness in the everyday. That is, space was carved out and set apart from ‘ordinary’ hospital environments to provide an arena for performing controlled ‘extraordinary’ patterns of action.
Sahin & Ozdemir 2016 (Turkey)	A general hospital	A descriptive survey. The Spirituality and Spiritual Care Rating Scale (SSCRS) (McSherry, Draper, and Kendrick, 2002). (17 items)	193 nurses	To investigate the nurses’ views to practicing spiritual care.	Data were analyzed using the SPSS for Windows, version 13.0. Descriptive statistics were used to describe nurses’ demographic characteristics.	All nurses participating were women (100%). Older nurses, married, higher levels of education, work experience, worked longer hours, received education in spiritual care, working in medical departments all tended to score higher on the SSCRS.
Tan et al., 2018 (Turkey)	Faculty of Medicine hospitals in 7 city centers (Toka, Ordu, Samsun, Elazıg, Van, Erzincan, Malatya) located in the Central Black Sea and Eastern Anatolian Regions.	A descriptive study. The Spirituality and Spiritual Care Rating Scale (SSCRS) (McSherry, Draper, and Kendrick, 2002). (17 items).	747 nurses	To explore Turkish nurses’ perceptions of spirituality and spiritual care and to investigate the relationship between their perceptions.	Data were collected by using a “Personal Information Form” and the “Spirituality and Spiritual Care Rating Scale” (SSCRS). Data were analyzed by using mean and percentage calculations in SPSS 16 package program.	The results of the study indicate that the knowledge of the nurses concerning spirituality and spiritual care was insufficient. It is thought that the spiritual aspect of the care services in both vocational education and in-service training should be examined.
